# The Universal Optimism of the Self-Evidencing Mind

**DOI:** 10.3390/e26060518

**Published:** 2024-06-17

**Authors:** Elizabeth L. Fisher, Jakob Hohwy

**Affiliations:** Monash Centre for Consciousness and Contemplative Studies, Monash University, Melbourne, VIC 3800, Australia; beth.fisher@monash.edu

**Keywords:** free-energy principle, active inference, self-evidencing, epistemology, wishful thinking, optimism bias, Friston, Machiavelli

## Abstract

Karl Friston’s free-energy principle casts agents as self-evidencing through active inference. This implies that decision-making, planning and information-seeking are, in a generic sense, ‘wishful’. We take an interdisciplinary perspective on this perplexing aspect of the free-energy principle and unpack the epistemological implications of wishful thinking under the free-energy principle. We use this epistemic framing to discuss the emergence of biases for self-evidencing agents. In particular, we argue that this elucidates an optimism bias as a foundational tenet of self-evidencing. We allude to a historical precursor to some of these themes, interestingly found in Machiavelli’s oeuvre, to contextualise the universal optimism of the free-energy principle.

## 1. Introduction: Active Inference and Wishful Thinking

The thoroughgoing conceptual motif in Karl Friston’s free-energy principle is *self-evidencing*—given the beliefs harnessed by an agent’s internal generative model, the agent selectively samples sensory data to maximise evidence for their own model. This means that agents select policies for action that they believe will reduce uncertainty given their model. Policies are selected in active inference, estimating which policy promises the best reduction in future uncertainty. Here, uncertainty should be understood in terms of the agent’s characteristic states, which are conveyed in the internal model as the states the agent expects to occupy.

Active inference, then, effectively appears to be an exercise in self-fulfilling prophesying. Crudely speaking, agents keep sampling until they acquire evidence for their beliefs about their expected states. If you ‘prophesy’ that you will be drinking coffee, then the possession of that prophecy induces a prediction error since you are not currently drinking coffee, and you then cast around sampling until the prediction error is quashed by observations of a hot cup of coffee in your hand.

Less crudely, actions can be in the service of either epistemic value or pragmatic value. In a particular context, agents can believe their best policy is to resolve uncertainty about the state of affairs—exploring to most efficiently update their model of which causes give rise to their observations (or what their preferences should be), or they can exploit, resolving uncertainty about their preferred state. Policies for utility and for epistemic value are selected on the basis of their ambiguity, the fidelity of the mapping from action to observation, and risk, the divergence between the expected observations under a policy and the expected states given the model. These quantities fall out of the free-energy principle, which then speaks directly to decision-making and resolves the exploration–exploitation dilemma for self-evidencing agents [1].

Equating inference with a broad concept of ‘thinking’, Friston and colleagues argue that this implies that all thinking is ‘wishful’, both thinking about fulfilling our preferences, such as drinking coffee, and thinking about information gain, such as looking in all the cupboards to find the coffee in the first instance [2]. We seek out the observations we wish for in active sensing and acting for utility. Notice that, in contrast to some colloquial understandings, the wishful thinking that accompanies self-evidencing under the free-energy principle is normatively neutral; one can wish for all sorts of things, positive or negative. You can wish for doom and gloom and go about selectively sampling observations to make that wish self-fulfilling.

The free-energy principle also underwrites the way agents balance accuracy and complexity. Agents strive to make accurate inferences, that is, they update their prior beliefs to acquire posterior beliefs that are most in accordance with the observations they sample. At the same time, agents need to minimise complexity, which is the divergence between their prior and posterior beliefs. Acquiring overly complex beliefs—new beliefs that diverge markedly from prior beliefs—bodes poorly for generalisability because they are likely to be overfitted and energetically and epistemically costly [1]. Self-evidencing agents, therefore, minimise free energy in ways that balance accuracy and complexity. Notice that, in active inference terms, accuracy sits with low ambiguity since ambiguous policies evidence beliefs poorly, and complexity sits with risk since complex model updates risk being far from the agent’s characteristic states.

## 2. The Epistemology of Active Inference

If all thinking is wishful (under the normatively neutral definition above), then there seems to be a threat to the epistemological aspects of belief formation under the free-energy principle. How can agents have justified beliefs and track reality if their belief formation is wishful? In order to assess this threat, we, therefore, turn to epistemology—the philosophical domain of theory of knowledge.

In epistemology, there are two main positions on justification of beliefs (for an overview, see [3]). According to *foundationalism*, beliefs are justified by basic beliefs that do not, themselves, require justification. According to *coherentism*, beliefs are justified by their relations to other, neighbouring beliefs in a web of belief. Self-evidencing aligns most obviously with coherentism because beliefs are indeed harboured in the internal model’s web of interconnected beliefs and because doubt is always resolved by an appeal to other beliefs about how to maximise information gain or minimise risk. Even hard-wired beliefs, such as a fish ‘believing’ it belongs in water, can be construed as self-evidenced over evolutionary time scales.

Concretely, the internal model of active inference agents embodies more or less explicit beliefs about the expected precision of evidence, which are used to control the information flow into and within the model. In other words, the agent keeps track of the precisions, at numerous time scales, of the sensory input and updates beliefs about precisions when they unexpectedly change (precision being the inverse of variance). The better the agent is at optimising its beliefs about precisions, the better they are at keeping track of volatility—non-linear changes—in their sensory input. An agent unable to learn such changes in variability will not change their learning rate or their habits, even when the world demands it (e.g., in response to increased variability due to illness or accident or, at a larger scale, due to severe weather events related to climate change, or the advent of new diseases such as COVID-19).

The internal model’s web of belief, therefore, encompasses both beliefs about states and causes in the world and metacognitive beliefs—beliefs about the precisions of other beliefs. Together, these beliefs drive the active sampling behaviours of the agent. If the sampled observations cohere with the expectations over the long-term average, then it is successfully self-evidencing.

There is a foundationalist tenor to self-evidencing as well. After all, everything the agent does is geared towards optimal sampling of observations that provide the most evidence for (that is, they justify) the agent’s beliefs; over time, beliefs are increasingly finessed through successive active sampling of observations. Prior beliefs will tend to acquire precision, and less and less updating will be necessary from observations unless volatile events occur requiring renewed learning and formation of new policies. This conforms to Bayesian inference, in particular empirical Bayes, where priors are dynamically determined by the evidential history of the model in question. If we assume that sensory evidence itself—as causal nudgings of the agent’s model—is independent of the web of belief, then the aspects of fundamentalism may come into place.

We, thus, regard it as a still-open question of how exactly self-evidencing is positioned in the debate between foundationalism and coherentism. There is, thus, some prospect that self-evidencing is an attractive epistemological framework because it may take what is best from both sides in this core philosophical debate (in epistemology, attempts in this vein are sometimes given the neologism ‘foundherentism’; cf. [4], a position that has been developed for predictive processing in [5]).

However, from the agent’s perspective, all that matters is the sampling of observations that cohere as best as possible with their beliefs in ways that the agent expects to be both accurate and unambiguous and neither too complex nor risky. Leaning into coherentism in this way implies that active inference agents are not directly or primarily concerned with seeking truth. As far as the agent is concerned, they need beliefs that are well-evidenced, given their model. That is, they are after beliefs that will let them occupy their characteristic states. If all those beliefs actually turn out to be false, then the agent need not care. In principle, the entire belief system can come adrift from truth and yet be highly coherent (for discussion, see [6,7]). For all the agent knows, everything can proceed swimmingly, yet in reality, they are a mere brain-in-vat, captured and fed phoney input from an evil scientist (cf. Cartesian scepticism).

Self-fulfilling prophesying, wishful thinking, unconcern with the truth, and scepticism might present active inference in an unfavourable, subjectivist, relativist light. The corrective to this picture is that the world needs to play its part too. Setting aside Matrix-like, evil scientist scenarios, self-evidencing will suffer if the wished-for observations are (literally) unrealistic. Your self-induced prediction error about having coffee in your hand will persist unresolved if there is no coffee around. And if acquiring coffee for some reason is unexpectedly risky (because the coffee bean wars have broken out), then persisting with the desire for coffee will lead to ruin. Wishful thinking is, in other words, bridled by reality. Hence, self-evidencing is not entirely and subjectively self-supervised; rather, though the agent is exclusively aiming for coherence, their inference is supervised by the world (and this position then appears to be a candidate foundherentism).

Zooming out, we can then make a distinction between the internal, generative model harboured by the agent and the external generative process, where states and their causes conspire to determine the agent’s sensory input. For example, the agent has beliefs about how to get coffee into the house and into the cup and into the mouth, and the world has facts about coffee, houses and mouths and facts about transitions among these states. Since we cannot rule out sceptical scenarios, there is no guarantee that the model and the process resemble each other—hence, we do not know if our beliefs track truth. However, an appeal to what is known as the *no-miracles argument* from the philosophy of science plausibly opens a door to realism. This is the idea that it would be a miracle if a scientific theory is predictively and instrumentally successful if it did not track truth to some degree; so, if we rule out miracles, then theories must track truth to some degree [8]. Similarly, if an agent manages to self-evidence, then its internal model must eventually begin to resemble the generative process, at least to some degree; it would be a miracle if it manages to persist in self-evidencing if its beliefs about how the world causes its sensory input were always wholly false. If there is no coffee, no coffee infrastructure, no cupboards, no coffee cups and so on, then it would be a miracle if acting on the belief that there are these things consistently gives you coffee observations.

Here, self-evidencing presents an intriguing dynamic. Given that the success of the generative model is not a miracle, it must stem from how the model begins to carry information about the generative process. This happens through the causal coupling of internal states of the model and the external states of the world, mediated through the sensory and active states of the organism’s boundary with the world (also known as the organism’s Markov blanket). Through active inference, this causal coupling runs both ways, not only from the world to the agent but also from the agent to the world. In other words, just as the internal model carries information about the world, the world begins to carry information about the agent, including the agent’s beliefs and preferences. This has implications for the Cartesian evil demon, who must be entrained by its victim’s beliefs and preferences if it wants the victim to exist (i.e., self-evidence) for any length of time (see [9]). It also has implications for non-sceptical environments, where agent’s actions can help shape the world so that the world comes to afford our wishful thinking (cf. also niche construction, see [10]).

Overall, active inference then implies a nuanced view of epistemology and realism, which removes the appearance that wishful thinking is a threat to knowledge. Though behaviour is driven by the agent’s beliefs, those beliefs are beholden to reality, and eventually, the agent’s beliefs seem likely to resemble reality. At least, the agent’s beliefs will recapitulate the structure of reality’s generative process, capturing the dynamic structure of how causes give rise to observations. The internal model represents the structure of the external world, with some degree of veridicality, but moulded from the perspective of the agent’s pragmatic concerns as they forage for information and utility. In this dynamic process, there will be increased coupled oscillation of the internal states of agents and the external states (cf. [9,11]), where the external world begins to carry information about—or “model”—the agent’s beliefs and preferences.

## 3. Biases Aplenty

Though the active inference agent’s web of beliefs is beholden to the evidence it can sample from the world, there is ample scope for false inference and misguided learning. Models are finessed over time, and more wholesale belief and policy revision and overall model selection are required when the agent encounters volatility. For example, you might spend much time experimenting before you learn to extract the optimal flavour from your expensive single-origin coffee beans, and then, you might have to forget it all and become a tea drinker when climate change begins to destroy quality coffee crops. In this unfolding, protracted (empirical Bayes) process, the agent can feasibly achieve intermittent, relatively satisfactory self-evidencing levels on the basis of biased beliefs (for example, enjoying coffee made with boiled water for a period of time based on the false belief that water temperature does not matter for coffee taste). Indeed, we should expect agents’ self-prophesying to have as many biases as reality allows at any given time.

In particular, if the agent’s starting point is wishful thinking (as discussed above), then false inference should be expected to be profligate, being only gradually whittled away by reality. Moreover, biases may well tend to persist longer than they need to because wishful agents will selectively sample observations that will confirm their beliefs. For example, it is often better for an agent to act to specifically confirm their biased beliefs about future outcomes than act to more unspecifically seek evidence that might lead to revision of those beliefs, since deviation of predictions from preferences comes with a risk cost [2].

Fundamentally, then, biases appear to be rational, since they can conform with Bayesian inference under the free-energy principle (cf. the complete class theorem [12]), and will persist as long as the agent’s selected observations manage to not contradict them sufficiently.

It seems that not all biases are created equally, since some afford better, more precise and robust self-evidencing over appropriate, relatively long timespans. You might have a bias that you are talented enough to be a professional sportsperson, which might serve you well in the short-term, making you try your best on the sports field, helping create social networks and staying healthy, but reality will soon enough crush that dream. In contrast, some perceptual biases seem to survive in the long-term probably because they facilitate mainly useful, if strictly false, inferences; an example might be when the ventriloquist effect illusorily makes sound appear from visible mouths when watching a movie on an older TV with offset speakers rather than being located where the speaker is. Of course, different kinds of biases are subject to different kinds of pragmatic and contextual factors and often reflect complex social dynamics that may give rise to undesirable biases, such as racial or gender biases.

Overall, we should expect active inference agents to manifest self-serving, wishful biases, which butt up against reality only to the degree agents cannot manage to make them self-fulfilling prophesies. And, of course, human agents are loaded with dozens, if not hundreds, of biases [13] (many of which we are blind to when it comes to ourselves [14]). Interestingly, in accordance with the active inference story we have told here, a recent reductive analysis sees biases as boiling down to belief plus belief-consistent (i.e., selectively sampled, wishful) information processing [15].

## 4. Optimism Bias and the Existence of Self-Evidencing Agents

Among the biases, some appear more robust and fundamental than others. The money illusion and the fame effect might be relatively socially and historically superficial, for example. In contrast, the optimism bias, which shall be our focus here, seems more widespread and general. To us, this suggests some biases may serve deep-seated purposes for our longer-term self-evidencing.

The optimism bias is a bias where our expectations are better than reality, such that agents overestimate the likelihood of good outcomes and underestimate the likelihood of bad outcomes [16]. For example, an optimistic agent may believe the chance of them having a good time at a party is higher than the likelihood actually is, or they might overestimate the likelihood of improving their health by starting a jogging habit, or they might believe the divorce rate for marriages is lower than the actual 50% [17]. The bias is observed in humans but also in a very wide range of other animals, such as goats, rats, pigs, mice, monkeys, chickens and other birds, hamsters, horses, dogs, lambs, cows, flies, sheep, bees and fish [18,19]. Note that the optimism bias, as it is discussed in this substantial body of literature, is precisely defined in terms of overestimation of the likelihood of good outcomes and underestimation of the likelihood of bad outcomes; hence, it is important to set aside more colloquial connotations of ‘optimism’. The optimism bias is typically assessed in individuals through a belief-updating task, where optimistic individuals update their beliefs more to positive than negative outcomes, or in ambiguous cue interpretation tasks, where individuals interpret ambiguous cues to indicate positive outcomes rather than negative outcomes.

The optimism bias has proven to be adaptive—helpful for self-evidencing—as individuals with optimism are associated with a range of better life outcomes from higher salaries at work to a reduced risk of cardiovascular disease [16,20,21]. The benefits from the optimism bias are in part due to how the bias promotes the agent to engage in the world; increased engagement in actions increases the chance of a positive outcome more than not engaging at all by minimising the risk of missed opportunity. For example, if an agent overestimates the likelihood of reducing their risk of cancer through diet and exercise, they will be more likely to both exercise and eat well. Although this may not achieve the reduced risk initially believed, the agent will be in a better position than if they were not to exercise or eat well at all.

Optimistic agents maintain their optimism bias by learning more from good news than bad news [22,23]. Asymmetric belief updating is one way optimism can persist in a world dominated by bad news; when optimists learn positive information, they will update the generative model underlying their decision-making more than when they learn negative information. Updating beliefs more to positive information creates a positive generative model, which tends to lead to good outcomes when there are sufficient good outcomes available in the generative process. Importantly, it follows that, in some contexts, the optimism bias can lead to maladaptive outcomes, namely where the context in question affords too few positive outcomes (for example, if the divorce rate was, in fact, 100%).

The context dependence of the optimism bias is relevant for its role with respect to self-evidencing. Research shows that optimism helped maintain immune responsiveness when exposed to a stressor [24]; however, when the stressor lasted more than one week, optimism was shown to be associated with a lower immune response compared to pessimism. This suggests that, as optimistic individuals engage more with a stressor, they may cope well for a small stressor that they can resolve and overcome, resulting in a positive immune response, whereas when the stressor is larger, they have the same engagement without the ability to overcome it, resulting in more stress and a weakened immune response. On the other hand, individuals who lack optimism do not engage with either type of stressor, resulting in a negative outcome for the mild stressor as they fail to overcome it when they could. Yet ignoring the large stressor can be a positive outcome as the individual is not constantly thinking and worrying about this issue (for discussion of this dynamic and depressive pessimism in the context of the free-energy principle, see [25]). This highlights the importance of the environment the agent is in for optimism to be beneficial. In other words, there needs to be chances for the agent to fulfil their optimistic belief in order for optimism to subserve optimal self-evidencing.

If the optimism bias is best characterised as enabling agents to minimise missed opportunities for minimising uncertainty, then that implies agents believe the world abounds with opportunity for self-evidencing, there for the taking. In this perspective, the optimism bias is not a bias in the sense of being irrational, since it simply reflects that the agent’s model includes a cost function (that is, a belief) that strongly favours seized opportunity over missed opportunity, even though seeking out opportunity can at times be costly. This would predict that optimists have less loss aversion, and that they tend to ignore losses more than wins.

**Figure 1:**
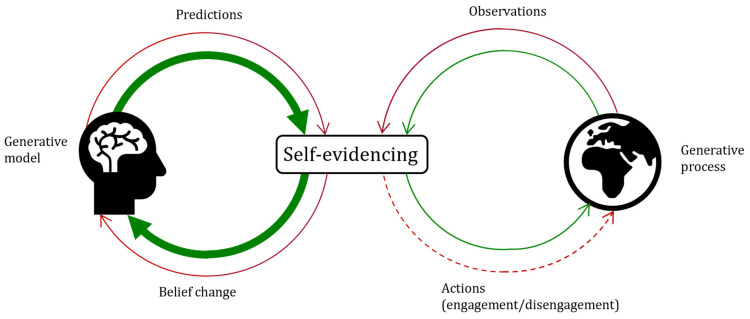

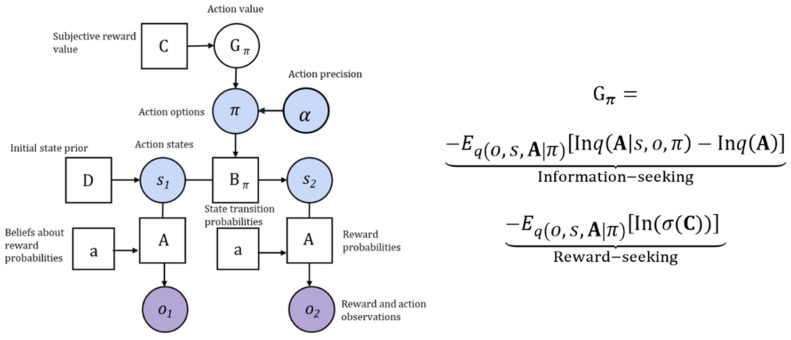
(**Top**) Self-evidencing involves both the generative model and the generative process. Figure 1 is a heuristic for optimistic self-evidencing. The agent has both stronger predictions about good outcomes and updates their beliefs more to good outcomes (green arrows, **left**). As the agent predicts and updates more to good outcomes, they select actions of engagement (optimistic, green arrow, **right**) rather than disengagement (pessimistic, red dashed arrow, **right**) with the world. The actions the agent selects affect the generative process such that their future observations can be dynamically influenced. Although optimistic self-evidencing is constrained by the generative process, the agent’s actions also influence the generative process through the dynamic coupled oscillation process described in the main text. One can imagine the same figure with a pessimistic agent whose predictions and belief updates are stronger for bad outcomes (i.e., a thick red arrow, **left**) and how this would lead to a different outcome in the dynamic process. (**Bottom**) A graphical depiction of an active inference model and the expected free-energy equation formalised in terms of information seeking and reward seeking. For details, see [26,27].

In fact, this is what we found in a study of optimism bias in rats, conceptualising the bias in terms of increased engagement and hypothesising that the animals would be more optimistic if treated with psilocybin [26]. Our further simulation modelling work with active inference adds to the face validity of this approach, capturing development, belief updating and behaviour with the same model of the bias [27]. This kind of work, thus, supports the overall validity of active inference models of the optimism bias (see Figure 1).

However, even if the optimism bias can be modelled in active inference and cast as rational (given a belief about minimising missed opportunity), a more complex picture emerges when viewed in the broadest possible terms of the free-energy principle. Given the increase in entropy over time—prescribed by the second law of thermodynamics—the universe is moving toward more disorder. This means that thermodynamic fluctuations are tugging at our (Markov blanket) boundaries at all times [11]. Concretely, this means that, every time an agent has arrived at a belief about something on the basis of their self-evidencing, they will learn that this is a kind of pyrrhic victory, since dissipative forces will soon undermine that belief and turn the positive outcome into a negative one that is less consistent with the agent’s belief. Put differently, as the current posterior becomes the prior for the next inference, agents should come to appreciate that all priors decline over time, mandating new belief formation (previously, we have leveraged this idea in conceptual and active inference models of binocular rivalry [28,29]). This persistent occurrence of negative outcomes suggests, at least initially, that, over appropriate time horizons, we really should be pessimists. Our opportunities for self-evidencing are, after all, limited by all the uncertainty we encounter in the shape of dissipative forces that, in the end, will literally tear us apart (that is, irreparably damage our organismic boundaries or Markov blankets [30]). No one lives forever, and societies and species vanish, having existed only as tiny blips on the radar of the universe. Puzzlingly, in spite of this *prima facie* case for pessimism, we, and many animals, are staunchly optimistic.

Appealing to the free-energy principle can help address this puzzle. We have primarily cast the free-energy principle in epistemic terms of self-evidencing, but this emerges out of an analysis of existence consistent with thermodynamics, such that organisms that exist must be self-evidencing [31]. Inexistence is conceived as having no boundary (or Markov blanket) and, therefore, what would be the agent’s internal states are, instead, dissipated through all possible states. Existence, in contrast, implies having an intact boundary and occupying only a limited set of states, namely those states that are characteristic for the organism, given its internal model (cf. homeostatic set ranges in biological organisms, which are expected—preferred—with high precision). To exist is to occupy a non-equilibrium steady state, appearing to transiently resist thermodynamic fluctuations. The existence of biological organisms, then, implies the ensuing active sampling of states (or observations at the boundary) that cohere with the internal model, which is how we have described self-evidencing within active inference.

According to this decidedly ‘high-road’ account of self-evidencing [32], an existing agent has a simple belief at the highest level of its hierarchy, namely “I exist”. This belief is equivalent to the agent’s belief that they self-evidence. That is, and against the cosmic odds, the agent believes that there are opportunities for reducible, explorable and exploitable uncertainty. Conversely, ceasing to believe that there are such opportunities for self-evidencing is for the agent to cease believing that they exist.

It may be, therefore, that the optimism bias observed in the everyday behaviour of many species is an expression of the fundamental tenet of the free-energy principle, namely that our existence is inextricably bound up with a belief that the world benevolently affords our existence—that the world offers us opportunities to seize in spite of its consistently observed inhospitality. This might be regarded as instrumentally rational because it keeps us going in the medium term, but on a more cosmic time scale, it does begin to look like wishful thinking in the pejorative sense.

This line of reasoning is already evident in the active inference literature:

Importantly, the agent’s generative model cannot simply mimic external dynamics (otherwise the agent would simply follow external dissipative dynamics). Rather, the model must also specify the preferred conditions for the agent’s existence, or the regions of states that the agent has to visit to maintain its existence or satisfy the criteria for its existence in terms of occupying characteristic states. These preferred states (or observations) can be specified as the priors of the model—which implies that the model implicitly assumes that its preferred (prior) sensations are more likely to occur (i.e., are less surprising) if it satisfies the criteria for existence. This means it has an implicit *optimism bias*. This optimism bias is necessary for the agent to go beyond the mere duplication of external dynamics to prescribe active states that underwrite its preferred or characteristic states [1], p. 46 (see also [33]).

Here, it will be useful to contrast the discussion so far with some of the literature on the free-energy principle where a different notion of ‘optimism’ is in play. In this literature, ‘optimism’ seems to be a label for wishful thinking (of the sort we described at the beginning of this paper). We do not believe using ‘optimism’ in that manner is apt, since one can wish for pessimistic outcomes. This appeal to optimism was spurred by one of the recent recurrences of the Dark Room Problem, which is the complaint that an agent seeking to minimise uncertainty should occupy a certain environment, such as a dark room. This complaint is heard often and repeatedly but is easily dismissed by invoking self-evidencing relative to a model that may or may not specify darkened rooms as characteristic. In the recent recurrence of the problem, a response was that agents leave the certain and, therefore, uninteresting dark room because they optimistically believe there will be information and utility on the outside (see response and replies in [34,35]). The problem with this response is that the agent who seeks out dark rooms in the first place would also do so because they ‘optimistically’ expect to occupy their characteristic states by sitting in the dark—but seeking out uninteresting states is not optimistic. A better way to tell the story about leaving the dark room emerges from the way we have introduced the optimism bias: the agent in the dark room will have decreasing priors driven by a belief in increasing volatility. Hence, over time, they will come to experience the dark room as increasingly uncertain, and then, they will leave in order to restore self-evidencing. They leave because they know deep down that dissipative forces will eventually get to them, and they optimistically believe that moving somewhere else may keep them safe for a bit longer—until they must move again. This is an optimism bias because agents go through life experiencing many losses (dissipative forces tend to emerge everywhere), and yet, they asymmetrically update their beliefs more for the (fleeting) wins they do experience. This keeps them going.

To further substantiate this role for optimism, we can appeal to the coupled oscillation implied by active inference (discussed above in the context of Cartesian scepticism). If agents act on their internal model’s optimistic belief, then the external states will begin to carry information—conform to—that belief. That is, by acting on the belief that the world benevolently offers opportunities to seize, the world will be shaped accordingly, facilitating self-evidencing. Consider how people pursue romantic partners based on the biased optimistic belief that the divorce rate is 10% rather than the actual rate of approximately 50%; acting on the bias does not deflate the divorce rate itself, but when enough of us are engaged, it does help orchestrate practices and communities so we keep congregating in various beneficial ways that would not occur if we all stayed home (that is, by acting on the optimistic belief, we help make it the case that the divorce rate does not plummet to 0% due to the fact that no one can be bothered to marry). We can also consider how, in spite of the dire planetary outlook from global warming, we nevertheless optimistically engage in mitigating action, which just might change the way global warming unfolds in ways that would not be possible if pessimism is allowed to reign. Put differently, by acting optimistically rather than realistically, we can potentially delay the time of the tipping point beyond which no action whatsoever can save us (for discussion, see e.g., [36], p. 489).

In short, self-evidencing entails the coupling of the generative model and generative process, which can help establish the universal optimism bias itself as a self-fulfilling prophesy, at least for some time. Here, it should be noted that coupling, as such, is neutral on existence and has the ability to withstand dissipative forces, such that the universal optimism bias implies a transient ‘master-slave’ dynamic, where the agent has enough of an upper hand in shaping the generative process—as long as the agent exists.

We have surmised that the optimism bias reflects the implications of the belief that we exist. We have placed this optimistically biased belief in our own existence somewhat uncomfortably between being rational and being irrational. We, thus, propose a *universal optimism bias*, which says that, from the perspective of the free-energy principle, agents are optimistic if they exist.

The behavioural manifestations of optimistically biased belief updating in humans and other animals could be rooted in other cognitive processes; so, our hypothesis about the origins of the optimism bias here is, of course, subject to empirical fortune. The hypothesis does make sense of the fact that optimism is associated with better health and life outcomes and is absent or reversed to pessimism in a range of life-shortening afflictions. If indeed the bias is universal, we should observe it across all active systems (including, perhaps, artificial machine-learning agents, cf. [37]). Since acting for utility and for epistemic value is balanced relative to the same objective function within the free-energy principle, we also predict that we will observe the optimism bias in epistemic action, such that agents update beliefs more after information gains than after information losses.

## 5. A Historical Perspective on Dissipative Forces and Optimism: Machiavelli

We want to end by broadening the perspective on the universal optimism bias by alluding to an intriguing historical precursor, namely Niccolò Machiavelli. This will help contextualise Friston’s work and infuse discussion of the themes coming to their apotheosis in the free-energy principle with a new and surprising historical ancestor.

It is a pity that, in the popular eye, Machiavelli is infamous for his blunt list of advice to autocratic rulers on how to brutally maintain power, set out in *The Prince*. Machiavelli also set out treatments of how to maintain republics, marked by civic liberty (where his real sympathies seem to lie), in *The Discourses,* and he discussed the objectives of militaries, in an argument against the instability caused by using mercenaries, in *The Art of War* (for an overview and references to Machiavelli’s works, see [38]). In our terms, Machiavelli articulated the requisite policies for action that predict low uncertainty and continued existence, given various generative models (e.g., of a prince, republic, or military).

The principle Machiavelli employs to write the very different sets of policies for these various generative models of governance rests on two opposing forces, *Fortuna* vs. *virtù*. *Fortuna* is not to be confused with our common notion of fortune as benign luck; *Fortuna* is cast by Machiavelli as a goddess of entropy, resembling “one of our destructive rivers which, when it is angry, turns the plains into lakes, throws down the trees and buildings, takes earth from one spot, puts it in another; everyone flees before the flood; everyone yields to its fury and nowhere can repel it” ([39]: Ch. 25). Likewise, *virtù* is not virtue in our conventional moralising sense but, rather, having a flexible disposition to vary behaviour according to Fortuna’s challenges. Therefore, “*Fortuna* shows her power where *virtù* and wisdom do not prepare to resist her, and directs her fury where she knows that no dykes or embankments are ready to hold her” ([39]: Ch. 25). Here, *virtù* is distinctively pre-emptive, keyed to anticipations of uncertainty and beliefs about how to hold it at bay. Based on this Homeric struggle between chaos and the policies for restraining it, Machiavelli indeed sets out sophisticated policies, relatively to particular generative models, that track notions of ambiguity and risk, exploration and exploitation, and accuracy and complexity.

Crucially, as everybody knows, against the will of the gods, mere humans always eventually lose. The resistance against *Fortuna* will, in the end, be futile, and yet, we optimistically persist. The flexible disposition of *virtù* rests on a belief that, this time, the dykes and embankments will hold against Fortuna; things will change so that it makes sense to engage after all. For example, the party tonight will be different from the many underwhelming past ones because, perhaps, tonight I will, in fact, be the social butterfly, or, at a larger scale, we optimistically engage in positive climate action to stem the (self-inflicted) forces of *Fortuna* felt worldwide. Machiavelli asserts in *The Discourses* the following:

[M]en are able to assist *Fortuna* but not to thwart her. They can weave her designs but cannot destroy them. They ought, then, never to give up as beaten, because, since they do not know her purpose and she goes through crooked and unknown roads, they can always hope, and hoping are not to give up, in whatever fortune and whatever affliction they may be ([40], p. 408).

With this Machiavellian framing, the brain is, therefore, marked with universal optimism as it enacts its *virtù*—its policies that it expects will maintain itself against the divine odds in an uncertain, ever-changing world.

## 6. Concluding Remarks

We have presented some of the core tenets of Karl Friston’s free-energy principle, pointing to its reliance on a universal optimism bias. We hope this articulation, which can be framed usefully in terms—presaged by Machiavelli—of dissipative forces and optimism, helps with placing this principle in our fundamental understanding of agents like us and other animals.

The free-energy principle itself might be cast by some as rather Machiavellian in the narrow-minded, nefarious sense. Friston could be observed as letting the principle loose as an organising, subduing framework in the chaotic zoo of theories about brains, behaviours, systems and the existence of things. However, in our experience, Friston is not out to forcefully subdue competing theories and frameworks in the blunt manner of the *The Prince* nor is he after the short-term victories in the scientific skirmishes of *The Art of War*; rather, he is, in the style of the republic governance of *The Discourses*, more pursuing long-haul success, fully expecting all scientific denizens to maintain their theoretical liberties, but comfortable in the knowledge that, implicitly or explicitly, they all do conform to the free-energy principle.

Fittingly, this belief in the long-term success of the free-energy principle reveals Friston’s own optimism bias too. In the philosophy of science, the ‘pessimistic meta-induction’ concludes that, since all scientific theories through history have been overturned by new theories, we should pessimistically expect our current theories to also be overturned [41]. The future will show if Friston’s optimism is warranted and if the free-energy principle indeed is the scientific equivalent of the black swan.

## Data Availability

No new data were created or analyzed in this study. Data sharing is not applicable to this article.
